# Potential effect of spliceosome inhibition in small cell lung cancer irrespective of the MYC status

**DOI:** 10.1371/journal.pone.0172209

**Published:** 2017-02-13

**Authors:** Kenichi Suda, Leslie Rozeboom, Hui Yu, Kim Ellison, Christopher J. Rivard, Tetsuya Mitsudomi, Fred R. Hirsch

**Affiliations:** 1 Division of Medical Oncology, University of Colorado Anschutz Medical Campus, Aurora, Colorado, United States of America; 2 Division of Thoracic Surgery, Department of Surgery, Kindai University Faculty of Medicine, Osaka-Sayama, Japan; University of South Alabama Mitchell Cancer Institute, UNITED STATES

## Abstract

Small cell lung cancer (SCLC) is a highly aggressive malignancy with few therapeutic advances in the treatment in recent decades. Based on a recent study that identified the spliceosome as a therapeutic vulnerability in MYC-driven breast cancers, we evaluated the efficacy of a spliceosome inhibitor in SCLC cell lines and analyzed the correlation with MYC status. Among 23 SCLC cell lines examined, eight showed high MYC protein expression (> 80% positive cells) by immunohistochemistry (IHC), while 10 cell lines demonstrated no staining for MYC. The remaining five cell lines showed weak staining (< 40% positive cells). All four cell lines that were previously demonstrated to have *MYC* gene amplification were positive for MYC by IHC. Four cell lines with high MYC expression and four with low MYC expression were used in further analysis. A spliceosome inhibitor, pladienolide B, showed high efficacy (IC_50_ < 12nM) in all eight cell lines tested, irrespective of the MYC IHC or *MYC* gene amplification status. We observed that the four cell lines with higher sensitivity to the spliceosome inhibitor were established from patients with prior chemotherapy. Therefore we chronically treated H1048 cells, that were established from a treatment-naïve patient, with cisplatin for 4 weeks, and found that H1048-cisplatin treated cells became more sensitive to pladienolide B. In conclusion, our *in vitro* results indicate that spliceosome inhibitors would be promising molecular target drugs in SCLC irrespective of the MYC status, especially in the second-line settings after an effective front-line chemotherapy.

## Introduction

Small cell lung cancer (SCLC) accounts for about 15–20% of lung cancer diagnoses, and is one of the most aggressive type of cancer with high mortality. Despite a recent development of molecular targeted therapy in non-small cell lung cancers [1], there have been few therapeutic advances in the treatment of SCLC in recent decades.

The widely known genetic alterations in SCLC are inactivating mutation/deletion of TP53 and RB1 and amplification of *MYC* family genes [2–5]. Amplification of one of the *MYC* family genes, *MYC* (also known as *MYCC*), *MYCL*, or *MYCN* in a mutually exclusive manner, was recognized in SCLC two decades ago [5]. Oncogenic MYC drives the expression of a broad number of genes with diverse functions, resulting in an increase in cell biomass [6]. However, to date, direct inhibition of the oncogenic transcriptional activity of MYC has been challenging to achieve [7].

A recent study found that the spliceosome, a dynamic macromolecular ribonucleoprotein (RNP) complex that catalyses the splicing of nuclear pre-mRNA into mRNA, is a therapeutic vulnerability in breast cancer models driven by MYC, due to MYC-induced elevation of mRNA synthesis [8]. To evaluate if this finding also applies to SCLC with MYC activation, we performed the current study to analyze the effect of a spliceosome inhibitor in SCLC cell lines with/without *MYC* gene amplification or MYC overexpression.

## Materials and methods

### SCLC cell lines and reagents

A total of 23 human SCLC cell lines were used in this study. All cell lines were in our archive or a kind gift from our collaborators. The short tandem repeat profiles of all cell lines used have been confirmed. All cells were cultured in RPMI1640 medium supplemented with 10% fetal bovine serum and 1x penicillin / streptomycin solution (Mediatech, Inc., Manassas, VA). Cells were grown at 37°C with 5% CO_2_ in a cell culture incubator. A spliceosome inhibitor, Pladienolide B, was purchased from Santa Cruz Biotechnology, Inc. (Dallas, TX). Cisplatin was purchased from Selleck Chemicals (Houston, TX).

### TMA preparation, antibodies and Immunohistochemistry (IHC) analysis

Formalin-fixed paraffin-embedded (FFPE) cell blocks were prepared to make a cell line tissue microarray (TMA). The TMA was sectioned at a thickness of 4 μm, and mounted on charged glass slides. MYC IHC staining was performed on a Ventana Discovery Ultra autostainer employing a c-MYC rabbit monoclonal antibody (clone Y69, Ventana). MYC staining was assessed by the study pathologist (H.Y.) using the H-score assessment which combines staining intensity (0–3) and the percentage of positive cells (0–100%).

### Cell proliferation assay

Cell proliferation was measured using the PrestoBlue Cell Viability Reagent (Life Technologies, Frederick, MD), according to the manufacturer’s instructions. Briefly, tumor cells (3 x 10^3^) were plated into each well of 96-well flat-bottomed plates and cultured for 24 hours. Pladienolide B, cisplatin, or dimethyl sulfoxide (DMSO) was added to the indicated drug concentration, and cells were incubated for an additional 72 hours. A colorimetric activity assay was performed by addition of the PrestoBlue reagent to each well and the plates incubated at 37°C followed by fluorescence detection (560nm Ex / 590nm Em) using a Biotek Synergy II plate reader. Percent growth was calculated relative to DMSO-treated controls. Statistical differences between growth curves was assessed using the ‘compareGrowthCurves’ function of the statmod software package (http://bioinf.wehi.edu.au/software/compareCurves).

### Establishment of cisplatin-treated cells

There are several methods to establish drug resistant cells, including continuous exposures to drug(s) [9–11] or the intermittent drug treatment that resembles “chemotherapy cycles” [12]. In this study, we used the former because parental cells were relatively tolerant to short-term cisplatin treatment. Cisplatin-treated cells were developed by chronic exposure to 5 uM concentration of cisplatin for 4 weeks for SW1271 and H1048 cells. Established cells were designated as SW1271/CDDP and H1048/CDDP cells, respectively.

## Results

### MYC expression in SCLC cell lines

**Fig 1:**
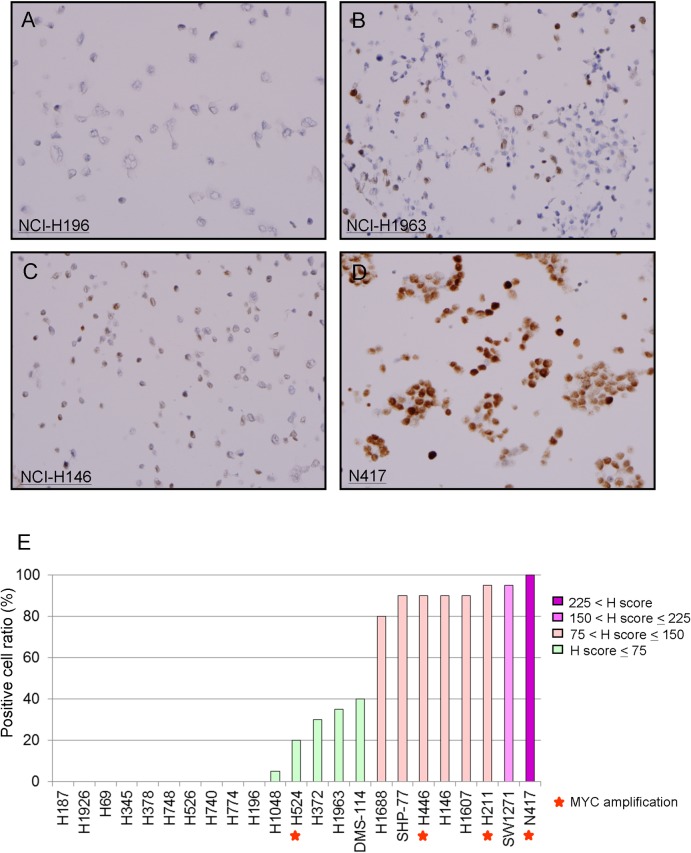
MYC expression in SCLC cell lines by IHC. Representative MYC IHC staining patterns are shown: *A*, negative (H196 / H-score 0); *B*, low MYC (H1963 / H-score 55); *C*, high MYC (H146 / H-score 140); and *D*, high MYC (N417 / H-score 230). *E*, summary of MYC IHC staining by positive cell ratio. The red star indicates cells with *MYC* gene amplification.

Representative MYC IHC staining patterns are shown in Fig 1A–1D. Of the 23 SCLC cell lines, which include four that were previously demonstrated to have *MYC* gene amplification [5], thirteen showed positive staining for MYC protein by IHC, while ten showed no staining for MYC (Fig 1E). MYC positive cell lines were divided into two groups based on positive staining of cells: eight cell lines with ≥ 80% positivity (high MYC) and 5 cell lines with < 40% positivity (low MYC).

### Efficacy of spliceosome inhibition in cell lines with different MYC status

**Fig 2:**
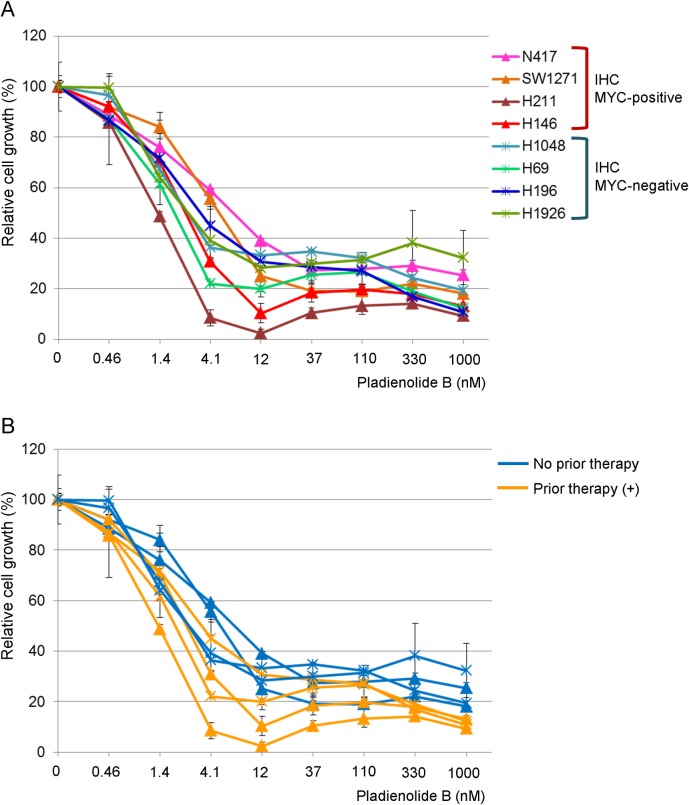
Efficacy of a spliceosome inhibitor, Pladienolide B, on SCLC cell lines. Cells were treated with Pladienolide B for 72 hours and the percent growth was calculated relative to the DMSO-treated control. *A*, cell lines were classified based on the IHC MYC expression status. *B*, cell lines were classified based on the presence / absence of a previous chemotherapy at the time of cell line establishment.

We then compared the growth inhibitory effect of a spliceosome inhibitor, Pladienolide B, in the high MYC group (N417, SW1271, H211, and H146 cells) and negative/weak MYC group (H69, H196, H1926, and H1048 cells). As shown in Fig 2A, both groups of SCLC cell lines, irrespective of either MYC IHC status or *MYC* gene amplification status, showed high sensitivity to Pladienolide B with an IC_50_ value of less than 12 nM. There was no statistical difference between the high MYC group and negative/weak MYC group (p = 0.32).

### Sensitivity of Pladienolide B and treatment history with chemotherapies

Because many of the SCLC cell lines were established from patients who had received chemotherapy, we divided these 8 cells lines into chemotherapy-naïve cell lines and those with prior chemotherapy based on the information written in the original papers [5,13,14]. We observed that the four cell lines with higher sensitivity to Pladienolide B were established from patients with prior chemotherapy, and this difference was statistically significant (p = 0.029; Fig 2B).

**Fig 3:**
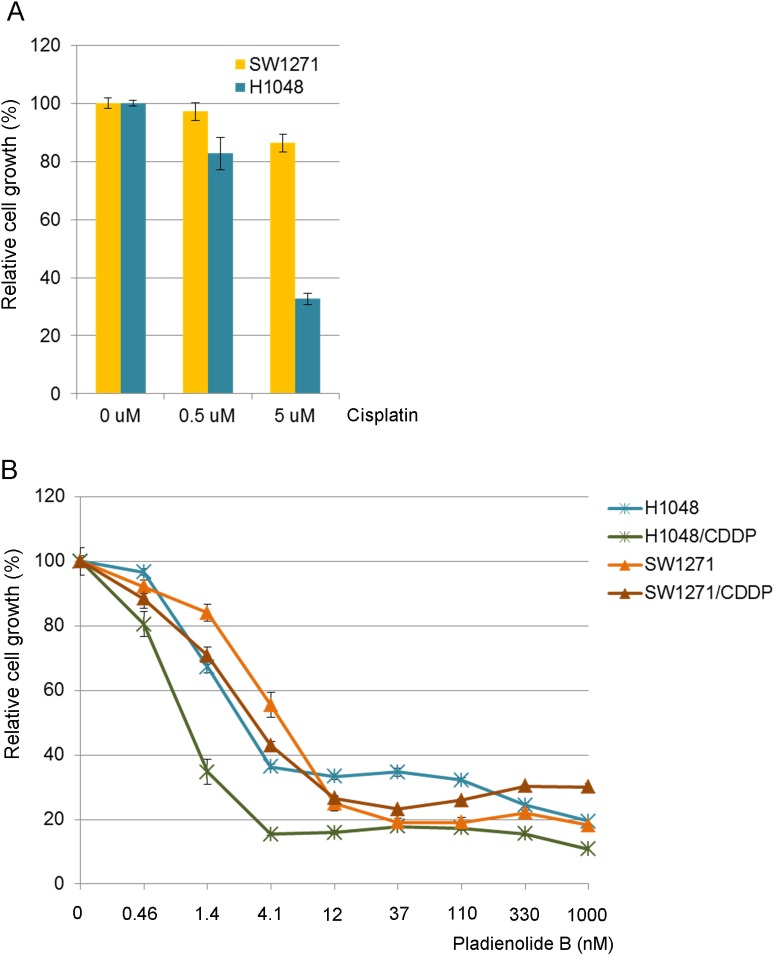
Establishment of cisplatin treated cells and the sensitivity to Pladienolide B. *A*, efficacy of cisplatin in SW1271 and H1048 parental cells. *B*, efficacy of Pladienolide B in parental cells and cisplatin-treated cells. Percent growth was calculated relative to DMSO-treated control.

Therefore we chronically treated two cell lines (SW1271 and H1048 cells), that were established from treatment-naïve patients, with 5 uM of cisplatin for four weeks. The two cell lines were choses as they grow adherent (suspension cells are difficult to select viable cells during drug treatment). H1048 parental cells were highly sensitive to cisplatin, while SW1271 cells were not (Fig 3A). Although SW1271 cells treated with cisplatin showed a very similar killing curve as compared to the parental cells against Pladienolide B, as we anticipated, H1048 cells treated with cisplatin became more sensitive to the spliceosome inhibitor following treatment (Fig 3B).

## Discussion

Deregulation of MYC has long been recognized as playing a role in human malignancies, including lung, breast, colon, and prostate cancers, Burkitt’s lymphoma and other hematological malignancies [15]. However, no targeted therapy has been clinically established to treat diseases with MYC aberrations. One reason for the failure to directly target MYC, at least in SCLCs, is that, unlike the epidermal growth factor receptor mutations or anaplastic lymphoma kinase rearrangement in lung adenocarcinomas, MYC de-regulation is not a sole driver aberration. For example, in mice models, functional inactivation of TP53 together with RB1 is sufficient for the development of SCLC, and MYC family member aberration occurs during SCLC progression [16]. Therefore, several recent studies have focused on indirect inhibition of oncogenic MYC activity using a synthetic lethal approach in targeting drugable proteins that are essential for the viability of MYC activated tumor cells but not for cells without MYC activation (a synthetic lethal approach). These efforts have identified several molecular target candidates such as SUMO-activating enzyme [17], PRKDC [18], THZ1 [19], FBXW7 [20], BUD31 and spliceosome components [8], and PIM1 kinase [21].

Among these candidates, we focused on the spliceosome inhibitor based on the correlation of spliceosome inhibition and MYC activation as reported in breast cancer [8], lymphomagenesis [22], and glioblastoma stem cells which overexpress MYC [23], but has not as yet been examined in SCLC. Targeting the spliceosome has been evaluated outside of the MYC [24–26], and the results of phase I clinical trials of a spliceosome inhibitor, E7107, has already been published [27,28], although these trials did not include patients with SCLCs.

We first examined MYC protein expression status in SCLC cell lines, because high MYC protein expression was also reportedly observed in non-*MYC* gene amplified cases from the analysis for SCLC patient specimens [29]. Subsequently we evaluated the efficacy of a spliceosome inhibitor, Pladienolide B, and found that the drug was effective in all SCLC cell lines (IC_50_ < 12nM) irrespective of the MYC protein expression or *MYC* gene amplification status. We consider that this is likely due to high mRNA synthesis in SCLCs irrespective of MYC status. Of interest is a recent study that evaluated Omomyc, a MYC dominant negative, in a panel of SCLC cell lines. This study observed that Omomyc suppressed the growth of all cell lines tested irrespective of MYC status [30].

Our results indicate that prior-chemotherapy affects the efficacy of the spliceosome inhibitor. The molecular background of this phenomenon is unclear, however we consider that cytotoxic chemotherapies may select cancer cells with higher mRNA synthesis. Long-term cisplatin treatment itself did not alter the efficacy of Pladienolide B in SW1271 cells with inherent cisplatin resistance, and this fact supports the above hypothesis.

In conclusion, our *in vitro* results indicate that spliceosome inhibitors would be promising molecular target therapy in SCLC irrespective of the MYC status, especially in the second-line settings after an effective front-line chemotherapy.
